# Small-cell neuroendocrine carcinoma arising from an extra-hepatic bile duct: a case report

**DOI:** 10.1093/gastro/goaa051

**Published:** 2020-10-21

**Authors:** Jae Ryong Shim, Jae Ri Kim, Youngmok Park, Hyung-Il Seo

**Affiliations:** 1 Division of HBP Surgery and Transplantation, Pusan National University Yangsan Hospital, Yangsan, Korea; 2 Department of Surgery, Biomedical Research Institute, Pusan National University Hospital, Gudeok-ro, Seo-gu, Busan, Korea

## Introduction

Neuroendocrine tumors (NETs) are neoplasms with variable malignant potential that may arise from neuroendocrine cells. The gastrointestinal tract is a common site for primary NETs and annually 1.3 for every 100,000 persons are newly detected [1, 2]. Although gastrointestinal NETs are common in the small bowel, appendix, rectum, and stomach, those that arise from the extra-hepatic bile duct are extremely rare (0.1%–0.4%) [3–5]. According to the 2010 World Health Organization (WHO) classification, NETs are classified as NET Grade 1 (mitotic count of <2 per 10 high power field [HPF] and/or ≤2% Ki-67 index), NET Grade 2 (mitotic count 2–20 per 10 HPF and/or 3%–20% Ki-67 index), NET Grade 3 (neuroendocrine carcinoma [NEC] includes small-cell carcinoma and large-cell carcinoma, mitotic count of >20 per 10 HPF and/or >20% Ki-67 index), and mixed adenoneuroendocrine carcinoma [6]. NECs originated from the extra-hepatic bile duct are extremely rare and the prognosis is very poor. These tumors are difficult to be diagnosed preoperatively because they do not present specific symptoms. The definitive diagnosis is usually confirmed through histopathological evaluations after a biopsy or surgery.

Here, we report the case of a 64-year-old man with jaundice who was diagnosed with small-cell NEC in the distal common bile duct (CBD).

## Case report

A 64-year-old man was referred from a local clinic with complaint of painless jaundice. The patient had past medical histories of diabetes mellitus, hypertension, chronic obstructive pulmonary disease, and gout. He had no surgical or family history of cancer.

**Figure 1.**
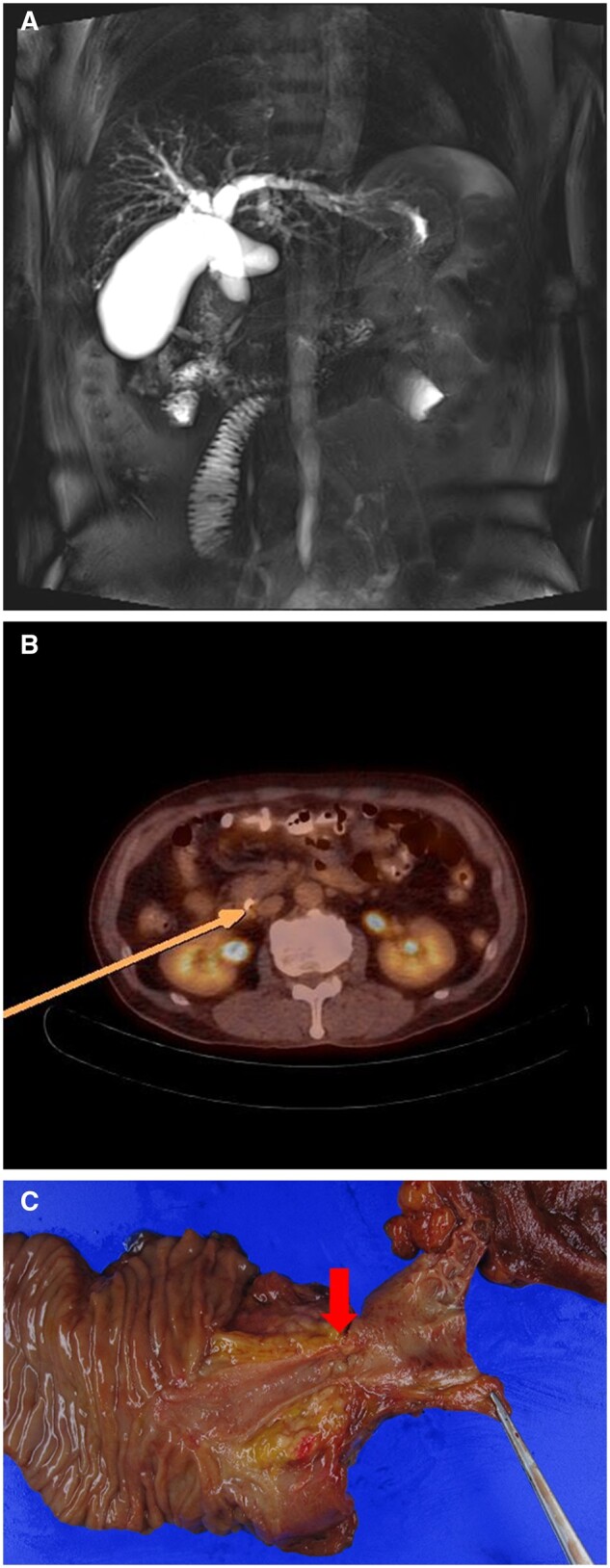
Radiologic findings and macroscopic appearance of the case of a small-cell neuroendocrine tumor in the distal common bile duct (CBD). (A) Magnetic response cholangiopancreatography (T2 projection image) shows the abrupt cut-off sign in distal CBD that strongly suggests cancer. (B) Positron emission tomography-computed tomography demonstrated the mild focal uptake (arrow, SUVmax 2.7) in the distal CBD. (C) The macroscopic appearance showed the narrowed lumen of the distal bile duct (arrow).

At admission, laboratory test results that present obstructive jaundice were as follows: total bilirubin 17.4 mg/dL, alkaline phosphatase 372 IU/L, carcinoembryonic antigen (CEA) 3.0 ng/mL, and carbohydrate antigen 19–9 235 U/mL. Magnetic response cholangiopancreatography revealed a focal wall thickening in the distal CBD and combined dilatation of the upstream bile duct (Figure 1A). Positron emission tomography-computed tomography showed a mild focal uptake (SUVmax 2.7) of the same lesion (Figure 1B). He underwent endoscopic retrograde cholangiopancreatography (ERCP) for preoperative biopsy and biliary drainage. Chronic inflammation with some atypical glands was detected by the biopsy. The immunohistochemical stain showed that tumor cells were positive for CK7 and p53 (weak) and negative for CK20 and CEA. The Ki-67 index was ∼20%.

The patient underwent pylorus-preserving pancreaticoduodenectomy (PPPD) under a presumptive diagnosis of distal CBD cancer. In the resected specimen, 2-cm tumor tissue was located in the distal CBD (Figure 1C). On microscopic examination, the tumor was seen to have invaded beyond the wall of the bile duct with perineural, lymphovascular invasion; however, no regional lymph-node metastasis was found among the 21 resected lymph nodes. The tumor cells had an increased nucleus-to-cytoplasm ratio. Mitotic counts were 20 cells per 10 HPF. In immunohistochemical stains, the tumor cells were positive for CD56, synaptophysin, and chromogranin A. The Ki-67 proliferative index was ∼60%. Therefore, according to the WHO 2010 guideline and the American Joint Committee on Cancer staging guidelines, this patient was diagnosed with T2N0M0 small-cell neuroendocrine carcinoma. He recovered without any abnormal events and was discharged in good condition on post-operative day 10. The patient received adjuvant chemotherapy with cisplatin/etoposide and had no recurrence during a follow-up of 36 months.

## Discussion

NECs in the biliary system are very rare [5, 7, 8]. The most frequent sites of NETs in extra-hepatic bile ducts are distal (19.2%), followed by middle (17.9%), cystic ducts (16.7%), and the proximal portion (11.5%) [9]. According to the 2010 WHO guideline, NECs are subcategorized as small-cell neuroendocrine carcinoma (SCC) and large-cell neuroendocrine carcinoma (LCC).

To identify the characteristics of tumors and the clinical features, we reviewed the literature, in which 21 cases including our case were reported about the SCC from 2000 (Supplementary Table 1). All 21 cases were based on the resected specimen after surgery. In the final pathologic report, the median tumor size was 3 cm (range, 0.3–6.2 cm) and lymph-node metastasis was observed in nine cases (42.9%). During the follow-up periods, five patients received adjuvant chemotherapy with various regimens (cisplatin with etoposide, irinotecan with carboplatin, etc.). Among the 15 cases with recurrence, the liver was the most common site of recurrence (38.1%) and the recurrence-free survival was expected to be <1 year. Although 4 cases did not report sufficient survival data, the prognosis for these 15 recurred cases was predicted to be poor and that of the remaining 6 patients without any recurrence was relatively good. Albores-Saavedra *et al.* [7] reported that 17 patients with SCC in an extra-hepatic bile duct died within 1 year after diagnosis.

Despite the development of the imaging studies, the preoperative diagnosis of NEC is still difficult. Especially, cholangiocarcinoma presents very similar characteristics and morphology compared with NEC with various modalities including ultrasound, computed tomography, or magnetic resonance imaging. In the present study, the patient was also diagnosed with distal CBD cancer in preoperative imaging studies. Although a preoperative tissue confirmation through ERCP could be helpful, the risks of false-negative results always exist. Moreover, preoperative biopsy does not tell us whether the tumor is SCC or LCC. Measurement of serum chromogranin A is helpful to diagnose NET [10]. Chromogranin A is known to be elevated in 90% of gut NETs and it is associated with the tumor burden and presence of recurrence. Therefore, serum chromogranin A could be an effective biomarker for the diagnosis of NETs before surgery. However, it is not cost-effective because of the rarity of NECs in extra-hepatic bile ducts.

Although WHO made the formal classification of NECs in extra-hepatic bile ducts, still little is known about the effective treatment strategy for this tumor due to its rare incidence and difficult preoperative diagnosis. Compared with the poor prognosis of conventional cholangiocarcinoma, NECs in extra-hepatic bile ducts have various clinical courses and prognoses with or without recurrences. So far, previous studies have reported quite different results about the prognosis of this tumor. Therefore, further studies with large cohorts from multiple institutions are needed to identify the clinical characteristics, to predict the true prognosis, and to establish the optimal management guidelines for this rare tumor.

## Supplementary Data

Supplementary data is available at *Gastroenterology Report* online.

## Authors’ contributions

H.I.S., J.R.K., and Y.P. conceived the study and participated in its design and coordination. J.R.S. and H.I.S. drafted the manuscript. All authors read and approved the final manuscript.

## Funding

None.

## Supplementary Material

goaa051_Supplementary_Data
