# Supplementary material for: Small-cell neuroendocrine carcinoma arising from an extra-hepatic bile duct: a case report
Source: Gastroenterol Rep (Oxf). 2020 Oct 21;9(4):380–2. doi: 10.1093/gastro/goaa051 (PMC8460092; doi:10.1093/gastro/goaa051)
Supplement: goaa051_Supplementary_Data [file goaa051_supplementary_data.docx]

**Supplementary Table 1. Literature summary of small-cell neuroendocrine carcinoma of the extrahepatic bile duct**

| **No.** | **Year** | **Authors** | **Sex** | **Age** | **CEA** | **CA19-9** | **Site** | **LN meta** | **Size (cm)** | **mitosis** | **ki67** | **CTx regimen** | **F/U (POD)** | **Status** |
| --- | --- | --- | --- | --- | --- | --- | --- | --- | --- | --- | --- | --- | --- | --- |
| 1 | 2000 | Kim, et al.[1] | M | 64 | n/a | n/a | CBD | LN | 3 | n/a | n/a | n/a | 5 | dead |
| 2 | 2001 | Edakuni, et al.[2] | F | 82 | n/a | n/a | CBD | LN(3/21) | 6.2 | n/a | LI 9.6 | n/a | 45 | NED |
| 3 | 2003 | Kuraoka, et al.[3] | M | 75 | n/a | n/a | CBD | LN | 4.5 | n/a | >90 | n/a | n/a | n/a |
| 4 | 2003 | Hazama, et al.[4] | M | 60 | n/a | n/a | CBD | LN | 0.3 | n/a | n/a | n/a | 12 | dead |
| 5 | 2004 | Kim, et al.[5] | M | 57 | n/a | n/a | CBD | n/a | n/a | n/a | n/a | n/a | 9.6 | dead |
| 6 | 2004 | Park, et al.[6] | F | 60 | 2.1 | 111.7 | mCBD | n/a | 3 | n/a | n/a | n/a | 5 | dead |
| 7 | 2005 | Kaiho, et al.[7] | F | 66 | n/a | n/a | CBD | n/a | 3.5 | n/a | n/a | n/a | 8 | dead |
| 8 | 2005 | Thomas, et al.[8] | M | 54 | n/a | n/a | hilar/CBD | n/a | n/a | n/a | n/a | n/a | n/a | n/a |
| 9 | 2006 | Jeon, et al.[9] | M | 65 | n/a | 2300 | CBD | LN(2/5) | 2 | n/a | n/a | Etoposide + ifofamide + cisplatin | 12 | dead |
| 10 | 2009 | Okamura, et al.[10] | M | 62 | normal | normal | mCBD | n/a | 3 | n/a | n/a | Irrinotecan + cisplatin, etc | 20 | dead |
| 11 | 2009 | Cho, et al.[11] | F | 59 | normal | normal | mCBD | n/a | 3 | n/a | n/a | n/a | 6 | alive |
| 12 | 2011 | Masui, et al.^[[1]](#endnote-1)^[12] | M | 82 | normal | normal | mCBD | n/a | 2.5 | n/a | 30~40 | n/a | 6 | dead |
| 13 | 2012 | Linder, et al.[13] | M | 82 | normal | 403(<28) | CBD | LN(2/9) | 1.9 | n/a | n/a | n/a | 6 | NED |
| 14 | 2012 | Baek, et al.[14] | F | 79 | n/a | 75.9 | CBD | LN(2/4) | n/a | n/a | n/a | n/a | n/a | n/a |
| 15 | 2013 | Ninomiya, et al.[15] | F | 75 | n/a | n/a | CBD | n/a | n/a | n/a | n/a | n/a | 14 | NED |
| 16 | 2014 | Lee, et al.[16] | M | 75 | n/a | 68.8 | mCBD | n/a | 2 | n/a | n/a | n/a | 11 | NED |
| 17 | 2015 | Kihara, et al.[17] | F | 70 | normal | 47 | hilar | LN(2/8) | 5 | numerous | 70 | Irrinotecan + carboplatin | 10 | NED |
| 18 | 2015 | Aigner, et al.[18] | M | 61 | n/a | 41.1 | mCBD | n/a | 1.5 | n/a | 90 | Etopocide + cisplatin | n/a | n/a |
| 19 | 2018 | Zhang, et al.[19] | M | 62 | 10.2 | 1073.6 | hilar | LN(3/5) | 2 | n/a | >80 | n/a | 6 | dead |
| 20 | 2019 | Zhang, et al.[20] | F | 64 | 31.3 | 40.4 | CBD | n/a | 4.5 | n/a | >50 | n/a | 12 | dead |
| 21 | 2020 | Present case | M | 64 | 3 | 235 | mCBD | n/a | 2 | 20 | 60 | Cisplatin + etoposide | 36 | alive |

CEA, carcinoembryonic antigen; CA 19-9, carbohydrate antigen 19-9; LN, lymph node; CTx, chemotherapy; F/U, follow up; POD, postoperative day; n/a, not available; CBD, common bile duct; mCBD, middle common bile duct; NED, no evidence of disease.

1. 1. Kim SH, Park YN, Yoon DS, et al. Composite neuroendocrine and adenocarcinoma of the common bile duct associated with Clonorchis sinensis: a case report. Hepatogastroenterology 2000;47:942-4

   2. Edakuni G, Sasatomi E, Satoh T, et al. Composite glandular-endocrine cell carcinoma of the common bile duct. Pathol Int 2001;51:487-90

   3. Kuraoka K, Taniyama K, Fujitaka T, et al. Small cell carcinoma of the extrahepatic bile duct: case report and immunohistochemical analysis. Pathol Int 2003;53:887-91

   4. Hazama K, Suzuki Y, Takahashi M, et al. Primary small cell carcinoma of the common bile duct, in which surgical treatment was performed after neoadjuvant chemotherapy: report of a case. Surg Today 2003;33:870-2

   5. Kim JH, Lee SH, Park J, et al. Extrapulmonary small-cell carcinoma: a single-institution experience. Jpn J Clin Oncol 2004;34:250-4

   6. Park HW, Seo SH, Jang BK, et al. [A case of primary small cell carcinoma in the common bile duct]. Korean J Gastroenterol 2004;43:260-3

   7. Kaiho T, Tanaka T, Tsuchiya S, et al. A case of small cell carcinoma of the common bile duct. Hepatogastroenterology 2005;52:363-7

   8. Thomas NE, Burroughs FH, Ali SZ. Small-cell carcinoma of the extrahepatic bile duct and concurrent clonorchiasis. Diagn Cytopathol 2005;32:92-3

   9. Jeon WJ, Chae HB, Park SM, et al. [A case of primary small cell carcinoma arising from the common bile duct]. Korean J Gastroenterol 2006;48:438-42

   10. Okamura Y, Maeda A, Matsunaga K, et al. Small-cell carcinoma in the common bile duct treated with multidisciplinary management. J Hepatobiliary Pancreat Surg 2009;16:575-8

   11. Cho SB, Park SY, Joo YE. [Small cell carcinoma of extrahepatic bile duct presenting with hemobilia]. Korean J Gastroenterol 2009;54:186-90

   12. Masui T, Doi R, Kawaguchi Y, et al. Adenoendocrine cell carcinoma of the extrahepatic bile duct: a case report and review of the literature. Clin J Gastroenterol 2011;4:174-178

   13. Linder R, Dorfman T, Ben-Ishay O, et al. Mixed neuroendocrine tumor of the common bile duct. JOP 2013;14:71-3

   14. Baek IY, Chung YJ, Park MK, et al. A Case of Mixed Adenoneuroendocrine Carcinoma in Common Bile Duct. Korean J Pancreas Biliary Tract 2012;17:56-60

   15. Ninomiya R, Ozawa F, Mitsui T, et al. [A case of neuroendocrine small cell carcinoma of the common bile duct]. Gan To Kagaku Ryoho 2013;40:1765-7

   16. Lee SW, Lee IS, Cho YK, et al. A case of mixed adenoneuroendocrine carcinoma of the common bile duct: initially diagnosed as cholangiocarcinoma. Korean J Pathol 2014;48:445-8

   17. Kihara Y, Yokomizo H, Urata T, et al. A case report of primary neuroendocrine carcinoma of the perihilar bile duct. BMC Surg 2015;15:125

   18. Aigner B, Kornprat P, Schollnast H, et al. A Case of Focal Small-cell Neuroendocrine Carcinoma in the Vicinity of the Extrahepatic Bile Duct, Adjacent to an Extensive Biliary Intraepithelial Neoplasm: A Diagnostic Challenge with Major Clinical Implications. Anticancer Res 2015;35:4821-8

   19. Zhang L, Wan D, Bao L, et al. Neuroendocrine carcinoma in the extrahepatic biliary tract: A case report and literature review. Medicine (Baltimore) 2018;97:e11487

   20. Zhang L, Yang Z, Chen Q, et al. Mixed adenoendocrine carcinoma in the extrahepatic biliary tract: A case report and literature review. Oncol Lett 2019;18:1585-1596 [↑](#endnote-ref-1)
